# Effect of orally administered moxaverine on ocular haemodynamics

**DOI:** 10.1186/1471-2210-11-S2-A45

**Published:** 2011-09-05

**Authors:** Stefan Palkovits, Michael Lasta, Doreen Schmidl, Agnes Boltz, Reinhard Told, Semira Kaya, Gerhard Garhöfer, Leopold Schmetterer

**Affiliations:** 1 Department of Clinical Pharmacology, Medical University of Vienna, 1090 Vienna, Austria; 2 Department of Ophthalmology and Optometry, Medical University of Vienna, 1090 Vienna, Austria; 3 Center for Medical Physics and Biomedical Engineering, Medical University of Vienna, 1090 Vienna, Austria

## Background

Reduced ocular perfusion is related to several common eye diseases, like age-related macular degeneration, diabetic retinopathy or glaucoma. Moxaverine, a phosphodiesterase inhibitor, has a vasodilating effect on peripheral vessels and is therefore clinically used to increase blood flow. It was recently shown that a dose of 150 mg moxaverine administered intravenously increases retinal blood flow in healthy subjects and in patients with ocular diseases. The aim of this study was to investigate the effect of oral moxaverine on ocular blood flow.

## Methods

Sixteen healthy subjects were included in this randomized, double-blinded, placebo-controlled, two-way crossover study and two study days were scheduled. The subjects were randomized to receive either 900 mg moxaverine (p.o. in three equal doses at two-hour intervals) or placebo on the first study day. On the second study day the subjects were crossed over to the alternative treatment. Ocular haemodynamics were measured at baseline and 5 hours after the study drug administration. A Laser Doppler Flowmeter was used to measure the choroidal and optic nerve head blood flow. The blood velocities in the retrobulbar vessels were assessed using the Color Doppler Imaging.

## Results

No difference was found in the haemodynamic parameters (choroidal, optic nerve head and retrobulbar blood flow) between moxaverine and placebo group. The p values for the choroidal and optic nerve head blood flow were p = 0.52 and p = 0.54, respectively. Similarly, the values assessed with the Color Doppler Imaging also showed no significant difference. Additionally, moxaverine had no effect on choroidal, optic nerve head and retrobulbar blood flow.

## Conclusions

Our results show that orally administered moxaverine, in contrast to intravenous moxaverine, does not alter the ocular blood flow. This may be related to the low bioavailability of moxaverine after oral administration.

